# Potential Impact of a Paper About COVID-19 and Smoking on Twitter Users’ Attitudes Toward Smoking: Observational Study

**DOI:** 10.2196/25010

**Published:** 2021-06-15

**Authors:** Chunliang Tao, Destiny Diaz, Zidian Xie, Long Chen, Dongmei Li, Richard O’Connor

**Affiliations:** 1 Department of Electrical and Computer Engineering University of Rochester Rochester, NY United States; 2 Department of Health Behavior Roswell Park Comprehensive Cancer Center Buffalo, NY United States; 3 Department of Clinical and Translational Research University of Rochester Medical Center Rochester, NY United States; 4 Department of Computer Science University of Rochester Rochester, NY United States

**Keywords:** COVID-19, smoking, Twitter, infodemiology, infodemic, infoveillance, impact, attitude, perception, observational, social media, cross-sectional, dissemination, research

## Abstract

**Background:**

A cross-sectional study (Miyara et al, 2020) conducted by French researchers showed that the rate of current daily smoking was significantly lower in patients with COVID-19 than in the French general population, implying a potentially protective effect of smoking.

**Objective:**

We aimed to examine the dissemination of the Miyara et al study among Twitter users and whether a shift in their attitudes toward smoking occurred after its publication as preprint on April 21, 2020.

**Methods:**

Twitter posts were crawled between April 14 and May 4, 2020, by the Tweepy stream application programming interface, using a COVID-19–related keyword query. After filtering, the final 1929 tweets were classified into three groups: (1) tweets that were not related to the Miyara et al study before it was published, (2) tweets that were not related to Miyara et al study after it was published, and (3) tweets that were related to Miyara et al study after it was published. The attitudes toward smoking, as expressed in the tweets, were compared among the above three groups using multinomial logistic regression models in the statistical analysis software R (The R Foundation).

**Results:**

Temporal analysis showed a peak in the number of tweets discussing the results from the Miyara et al study right after its publication. Multinomial logistic regression models on sentiment scores showed that the proportion of negative attitudes toward smoking in tweets related to the Miyara et al study after it was published (17.07%) was significantly lower than the proportion in tweets that were not related to the Miyara et al study, either before (44/126, 34.9%; *P*<.001) or after the Miyara et al study was published (68/198, 34.3%; *P*<.001).

**Conclusions:**

The public’s attitude toward smoking shifted in a positive direction after the Miyara et al study found a lower incidence of COVID-19 cases among daily smokers.

## Introduction

### Background

COVID-19 is caused by SARS-CoV-2 [1], and given that it is mainly a disease of the respiratory tract, researchers have investigated whether cigarette smokers or vapers are at higher risk of SARS-CoV-2 infection, worse COVID-19 disease severity, worse clinical outcomes, or mortality. Although most literature shows that smoking worsens COVID-19, there is seemingly paradoxical evidence regarding this association. Smoking history appears to increase the risk of severe disease in hospitalized patients, particularly among younger patients without diabetes [2]. An increased risk of mortality has also been shown among current smokers [3]. On the other hand, SARS-CoV-2 binds the angiotensin-converting enzyme 2 (ACE2) receptor. While it is unclear whether smoking increases the level of ACE2 receptor expression in the respiratory tract, nicotine may also compete with SARS-CoV-2 for binding of the nicotinic acetylcholine receptor [4]. The interrelationship among smoking, nicotine, SARS-CoV-2, and COVID-19 is an active and evolving area of research, where new studies emerge regularly.

Researchers in France—Miyara et al—conducted a cross-sectional study on patients infected with COVID-19 in a large French university hospital, which was placed on a preprint server on April 21, 2020 [5]. The results showed that the rate of current daily smokers was significantly lower among outpatients and inpatients with COVID-19 (6.1% and 4.1%, respectively) as compared to that in the French general population after standardization by age and sex [5], which was estimated to be between 22.4% and 26.9% [6]. The authors concluded that their results suggest that active smokers may be protected against symptomatic COVID-19 [5]. However, they did note that health care workers were overrepresented in the outpatient group; patients in intensive care units were excluded; smoking status may have been under- or overreported; smoking status was assessed only in symptomatic patients with COVID-19, even though many infected individuals are asymptomatic; and the association found does not imply causality [5]. Furthermore, the authors of this paper emphasized that nicotine and the nicotinic receptor were of interest and acknowledged the negative consequences of smoking cigarettes [5]. A follow-up report suggested studying nicotine patches as a preventative option against COVID-19 [7]. Despite the limitations being noted in the paper, the title and nature of the main results could mislead the general public, who typically attend to headline findings and not caveats. This study on COVID-19 incidence among smokers was published in Qeios, an open science publishing platform, in May 2020. The article metrics on Qeios demonstrate that the paper was mentioned by one news outlet and mentioned directly by 126 tweets, 8% of which were in the United States, and has a top 5% attention score as measured by Altmetric.

Twitter, a *microblogging* platform [8], can contribute to scientific knowledge dissemination and translation [9]. Throughout the COVID-19 pandemic, Twitter has served as a platform for users to express their opinions, share information, and receive information from others—over 63 million English tweets worldwide used COVID-19–related keywords from January to July 2020 [10]. With the evidence arising from the Miyara et al study regarding the relationship between smoking and the novel coronavirus, conversations on Twitter about the study may provide an interesting case study in the transmission of potentially controversial or contrarian findings.

### Objective

A previous Twitter study on COVID-19 and smoking—the only other Twitter analysis on this topic, to our knowledge—showed that preprints suggesting the benefits of smoking might increase reactions to tweets on tobacco products and the virus [11]. During this pandemic, people may be looking for something they can do to lower their risk. Methods for reducing the spread of the virus, such as using masks and quarantine, and discussion of fear and stress due to the lack of preventative options were found to be popular topics among Twitter users [12]. There is a possibility that those looking for a preventative action against COVID-19 could use the Miyara et al study as a rationale to take up smoking or vaping or to delay quitting. This paper presents a novel view of the change in sentiment toward smoking *before and after a specific paper* *was published* suggesting that the incidence of COVID-19 was lower among smokers compared to the general population. In this report, we aim to examine the spread of the Miyara et al study among Twitter users, attitudes toward the study, attitudes toward smoking, and whether there was a shift in sentiment toward smoking and nicotine after April 21, 2020.

## Methods

### Data Collection and Preprocessing

**Figure 1:**
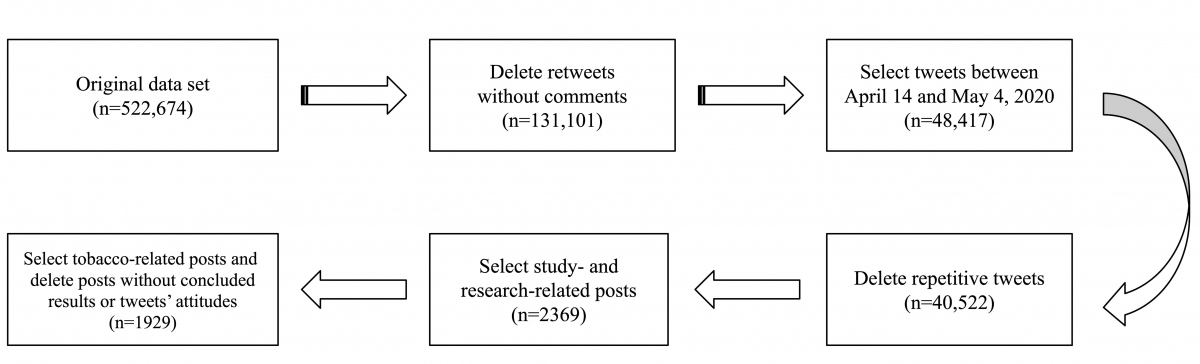
Data set preparation procedures.

The related tweets (ie, Twitter posts) posted from April 14 to May 4, 2020, were crawled by the Tweepy stream application programming interface using keyword queries with COVID-19–related keywords, including “CORONA,” “corona,” “COVID19,” “covid19,” “covid,” “coronavirus,” “Coronavirus,” “CoronaVirus,” and “NCOV.” The analysis period was chosen due to the nature of our study objective. Because we were interested in the change in sentiment before and after publication of the paper, we analyzed tweets that were posted immediately before and after the day it was published. Next, retweets without comments were deleted, since simple retweets typically do not explicitly reflect personal opinions; the behavior of retweeting can mean supportive, oppositional, or neutral attitudes toward the original tweet. Repetitive tweets were also removed from the collected data set, as the majority were copied news headlines without personal sentiments. Afterward, research- and tobacco-related tweets were filtered out in sequence using keyword matching: we first filtered research-related tweets using “study” and “research,” then tobacco-related tweets were filtered using “smok,” “cigarette,” “tobacco,” “nicotine,” and “ace2.” Tweets discussing studies without clear findings were removed as those having no impact in shifting people’s opinions. Finally, 1929 tobacco- and research-related tweets remained and discussed, in some way, the effects of smoking on COVID-19 infections and symptom development. Figure 1 shows the data preprocessing procedures for obtaining our final data set of 1929 tweets.

### Sentiment Analysis

Three categories were used to categorize each tweet: (1) whether it was about the Miyara et al study (yes or no), (2) the article’s attitude toward smoking (positive, negative, or neutral), and (3) the user’s attitude toward smoking as expressed in the tweet (positive, negative, or neutral). For the first category, whether or not each tweet was related to the published Miyara et al study was manually coded for all the selected tweets. For the second category, the article’s attitude toward smoking as discussed in the tweet was manually coded as positive, neutral, or negative. For example, if the tweet discussed an article that found smokers were at more risk for COVID-19, it was considered negative. For the third category, each tweet was manually coded as positive, neutral, or negative based on the user’s attitude toward smoking. For example, if the user said, “smoking is bad for you,” the tweet was categorized as negative. The term *smoking* referred to in this category was not necessarily used in regard to cigarettes and could refer to the use of other tobacco products as well.

Two coders from the study team coded each tweet separately and disagreements were discussed among team members to achieve final agreements. High agreements were achieved between the two independent coders on coding the 1931 selected tweets into different categories. The Cohen κ value was 0.92 (95% CI 0.90-0.94) for categorizing whether the tweet discussed the French study. Regarding the article’s attitude toward smoking, the Cohen κ was 0.93 (95% CI 0.92-0.95) for categorizing the attitude into positive, neutral, and negative groups, indicating very high agreement. The Cohen κ was also very high when categorizing the tweet’s attitude toward smoking into positive, neutral, and negative groups, with almost perfect agreement (κ=0.86, 95% CI 0.85-0.88).

### Statistical Analysis

According to whether the tweet was related to the Miyara et al study and whether the tweet was posted before or after the publication date of the Miyara et al study (ie, April 21, 2020), all selected tweets were classified into three groups: (1) tweets not related to the Miyara et al study before publication, (2) tweets not related to the Miyara et al study after publication, and (3) tweets related to the Miyara et al study after publication. The tweets’ attitudes toward smoking and the articles’ attitudes toward smoking were compared among the above three groups using multinomial logistic regression models through the *multinom* function in the *nnet* package in the statistical analysis software R, version 4.0.5 (The R Foundation). The significance level of all two-sided tests was set at 5%. The follower counts of posters within groups related and not related to the Miyara et al study were analyzed to reveal the impacts of when tweets were posted by these accounts.

### Topic Analysis

In order to capture certain themes that were prevalent within the tweets, the two members of the study team that hand-coded the tweets also created topics based on the content they read. The tweets were separated into two basic categories to allow for a more efficient comparison of themes: tweets that were not about the Miyara et al study and tweets that were about the Miyara et al study. Different focuses were adopted during the theme-capturing process, considering the fact that people react differently within these two groups. Specifically, themes from tweets about the Miyara et al study were mainly people’s attitudes toward the research itself or speculations about unseen driving forces. Contrary to a comparably narrow but concentrated scope, tweets that were not related to the Miyara et al study discussed diverse aspects of the field. For example, users showed sentiments beyond attitudes toward smoking, such as generally distrusting research, stating reasons to support their stances, and requesting information for truth finding. Such diversity has also been observed through various research directions that analyzed smokers’ risks of COVID-19 infections, which include but are not limited to the analysis of existing health conditions and harmful life habits. To obtain a comprehensive understanding of these themes, which are not necessarily correlated with each other, multiple groups are, thus, needed for illustrations. As shown in the Results section below (Tweets Related to the Miyara et al Study subsection), groups defined as *stances on smoking*, *other sentiments*, and *research focus on association between smoking and COVID-19* are used to reflect the uniqueness of sentiments. Based on the themes that were notably expressed most in the tweets, topics were created for each of the two categories: tweets that were not about the study and tweets that were about the study. The topics for both tweets that were about and that were not about the Miyara et al study were chosen by the manual coders, who noted recurring themes throughout the hand-coding process. Each tweet was then categorized under one of the topics or put under the category *miscellaneous* (ie, unsorted). For each topic, tweets were chosen as a representative example of other tweets within that topic.

## Results

### Temporal Analysis

**Figure 2:**
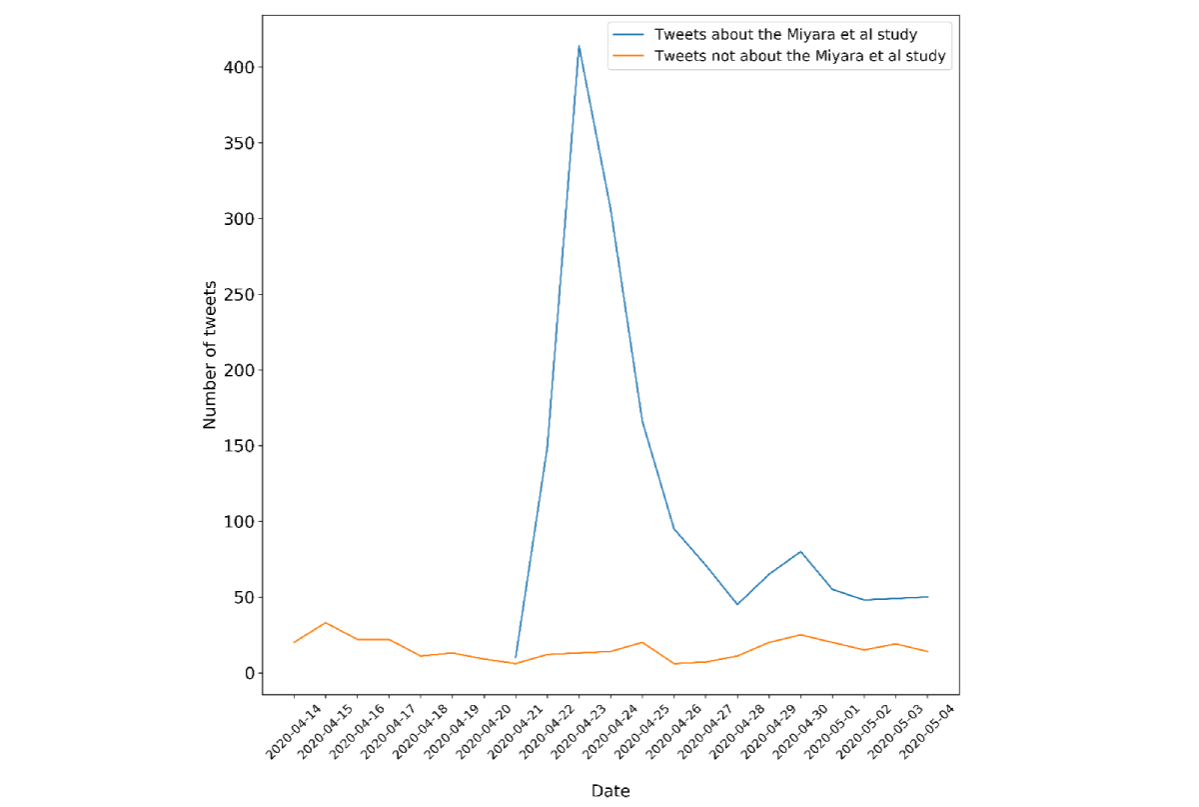
Temporal analysis of the Miyara et al (2020) study’s influence.

The temporal analysis of the Miyara et al study was done by compiling the hand-coding results after the sentiment analysis. A clear comparison between numbers of tweets that were about the Miyara et al study versus those that were not about the Miyara et al study can be drawn from Figure 2. As shown, tweets that were not related to the Miyara et al study remained relatively steady through the study period. In contrast, tweets related to the Miyara et al study sharply increased beginning on April 22, 2020, the day after publication, with a spreading peak observed between April 23 and 24, 2020, when discussion appeared to be most intense. Throughout the whole period until May 4, 2020, the number of tweets related to the Miyara et al study surpassed all other tobacco- and COVID-19–related tweets, confirming its prevalence on Twitter.

### Sentiment Analysis

**Figure 3:**
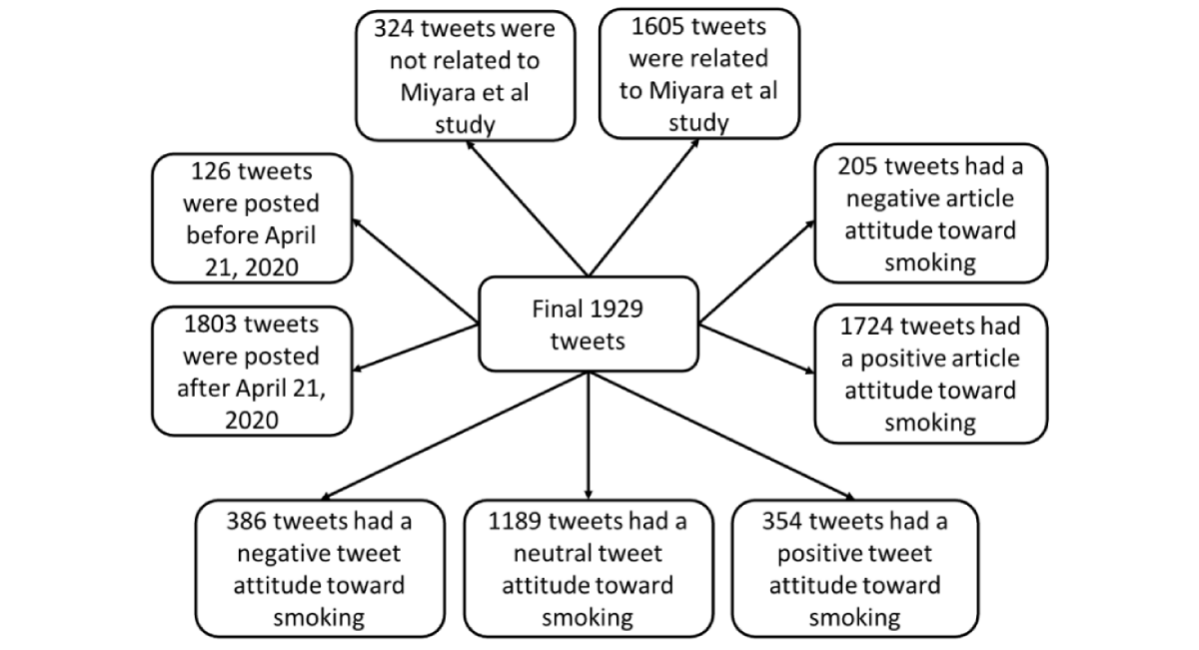
Final tweet classification into different categories.

Figure 3 shows the classification of the final 1929 tweets in different categories (Cohen κ ranged from 0.86 to 0.93). Among the 1929 selected tweets that cited articles with either positive or negative attitudes toward smoking, 324 tweets (16.80%) were not related to the Miyara et al study, while 1605 tweets (83.20%) were related to the Miyara et al study.

**Figure 4:**
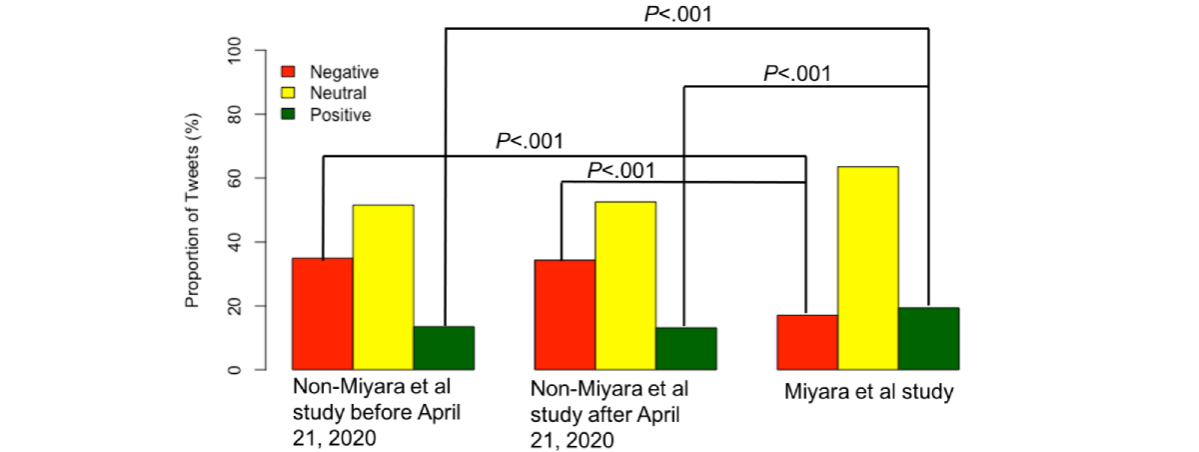
Proportion of negative, neutral, and positive tweets regarding their attitudes toward smoking in three different tweet groups: (1) tweets not related to the Miyara et al study (Non-Miyara et al) before April 21, 2020; (2) tweets not related to the Miyara et al study (Non-Miyara et al) after April 21, 2020; and (3) tweets related to the Miyara et al study. *P* values were obtained from the pairwise comparisons within the multinomial logistic regression model framework.

Figure 4 shows the proportions of negative, neutral, and positive tweets regarding their attitudes toward smoking in the three different tweet groups. In tweets not related to the Miyara et al study before April 21, 2020, 17 out of 126 tweets (13.5%) showed positive attitudes, 65 out of 126 tweets (51.6%) showed neutral attitudes, and 44 out of 126 tweets (34.9%) showed negative attitudes toward smoking. In tweets not related to the Miyara et al study after April 21, 2020, 26 out of 198 tweets (13.1%) showed positive attitudes, 104 out of 198 tweets (52.5%) showed neutral attitudes, and 68 out of 198 tweets (34.3%) showed negative attitudes toward smoking. In tweets related to the Miyara et al study, 311 out of 1605 tweets (19.38%) showed positive attitudes, 1020 out of 1605 tweets (63.55%) showed neutral attitudes, and 274 out of 1605 tweets (17.07%) showed negative attitudes toward smoking. Multinomial logistic regressions were conducted to compare the differences in proportions of positive and negative attitudes toward smoking across the three different groups. The proportion of tweets showing a negative attitude toward smoking that were not related to the Miyara et al study was significantly higher (*P*<.001) than the proportion of tweets showing a negative attitude toward smoking that were related to the Miyara et al study (*P*<.001). Meanwhile, the proportion of tweets showing a positive attitude toward smoking that were not related to the Miyara et al study was significantly lower (*P*<.001) than the proportion of tweets showing a negative attitude toward smoking that were related to the Miyara et al study (*P*<.001).

### Topic Analysis

#### Tweets That Were Not Related to the Miyara et al Study

For the 324 tweets that were not related to the Miyara et al study, 126 (38.9%) tweets were posted before April 21, 2020, and 198 tweets (61.1%) were posted on April 21 or after April 21, 2020. The 324 tweets were categorized into three groups of perspectives to analyze users’ opinions, various sentiments, and the research focus of the studies mentioned: *stance on smoking*, *other sentiments*, and *research focus on association between smoking and COVID-19*, respectively (Table 1). To better capture topic details, many posts were coded into more than one category. After comparison, similar weight distributions were seen between the two periods—before and after April 21, 2020—within the group *stance on smoking*, with the majority of tweets falling into the category *stating the finding*, followed by those within the categories *discourage tobacco* and *encourage tobacco*. Significant changes were found between the two periods in the weights within the categories *more info requested* (*P*=.006), *advocating quitting smoking* (*P*=.01), and *health conditions* (diabetes, asthma, etc) (*P*<.001) using the two-proportion *Z* test. With weight decreases of 6.99%, 6.80%, and 12.79%, their two-proportion *Z* test scores were 2.75, 2.47, and 4.56, respectively, which shows that there were statistical differences with a 5% confidence interval with respect to these three categories between the two periods. For other categories, no clear change was observed. Table 1 shows a breakdown of categories with two example tweets provided for each topic.

**Table 1:** Topic categories for tweets not related to the Miyara et al study.

Group and topics			Tweets before April 21, 2020 (n=126), n (%)		Tweets after April 21, 2020 (n=198), n (%)		Example tweets^a^
**Stance on smoking**							
	Stating the finding	58 (46.0)		97 (49.0)		“shocking result smokers are far less likely to be hospitalized with coronavirus than non smokers” “smoking is associated with doubling of covid 19 progression risk center for tobacco control research and education”	
	Discourage tobacco	39 (31.0)		67 (33.8)		“this study is simple and others published since have made it pretty clear smokers die more from covid than nonsmokers” “this suggests that there has never been a better time to quit smoking to protect yourself from covid 19 study shows an incredibly high correlation between serious covid symptoms and habitual smoking”	
	Encourage tobacco	13 (10.3)		23 (11.6)		“bibber indepth if you split current and former smokers current smokers will end up with a lower risk for hospitalization than or 0 7 and former smokers with a higher or there is no misleading this is a well done study of 4 103 corona patients in a nyc health system” “nicotin from tobacco can cure covid19 clinical trials are on in australia tobacco nicotin used as last remedy cure in medical science tobacco is best medicine for neurological disorder do some research goi is right”	
**Other sentiments**							
	Advocate quitting smoking	13 (10.3)		7 (3.5）		“a new study shows that cigarettes can help the coronavirus enter lung cells meaning it’s time to stop smoking” “correct in fact most research on vaping and respiratory health shows that smokers who quit by switching to vaping experience better lung function reduced pneumonia risk and less severe asthma and emphysema”	
	More info requested	12 (9.5)		5 (2.5)		“are there any official recording of those who smoke are more likely to suffer from covid19” “love your show do you have any more info about the study that said smokers are less likely to die from coronavirus thank you”	
	Debate with others	4 (3.2)		5 (2.5)		“kwaza really please reference one scientific study that proves that smoking decreases your chance of surviving a covid19 infection specifically a study by the cdc found that just over 1 of those who died from a covid19 infection were smokers and just over 2 were previous smokers” “ok this isn t being reported enough you are at risk of covid if you are obese have asthma and smoke on a regular basis it is your responsibility to do your own research and act accordingly you can literally get rid of these conditions in weeks if you act now”	
	General distrust in research	3 (2.4)		5 (2.5)		“such a musical chair this research on covid19 has become smokers will be at higher risk then smokers have a better chance of surviving this is when you shut it all off fda says smokers may have higher risk of catching covid 19” “those researchers based their recommendation against smoking on general attributes of the virus eg covid attacks the respiratory system it doesn t appear they consulted the covid specific data at all before issuing their advisory”	
**Research focus on association between smoking and COVID-19**							
	Smoking itself	97 (77.0)		185 (90.9)		“smoking does not increase covid 19 susceptibility” “smoking protects against covid 19 symptoms says new research hiptoro interesting given the need for oxygen uptake of covid 19 victims”	
	Health conditions	18 (14.3)		3 (1.5)		“only half of urban and rural pakistanis are aware of the fact that diabetics smokers and asthmatics are at a higher risk of falling severely ill from covid 19 study by aga khan university aku coronavirus” “it looks like the best chance to survive coronavirus is to be an overweight smoker according to the latest research justsaying”	
	ACE2^b^ related	11 (8.7)		12 (5.6)		“icilondres one theory is that nicotin blocks ace2 receptors the backdoor to the lungs for covid19 however research needs to be done before we all reach out to our patches” “some researchers suspect that nicotine binds to ace2 as well and that this makes i via”	

^a^During preprocessing and before hand-coding, punctuation and capitalization were removed from the tweets to facilitate content analysis.

^b^ACE2: angiotensin-converting enzyme 2.

#### Tweets Related to the Miyara et al Study

While manually coding the 1605 tweets about the Miyara et al study, certain common themes arose: encouraging tobacco use, expressing feelings of surprise about the findings of the Miyara et al study, stating the main finding of the Miyara et al study, discussing the follow-up nicotine patch study, highlighting the negative consequences of tobacco use, and questioning whether or not the Miyara et al study was funded by the tobacco industry. Table 2 shows a breakdown of these categorized tweets along with two example tweets per topic. There is a possibility of topic overlap of these tweets that has not been accounted for here. From the table, we can see that the most common theme was tweets that stated the main finding of the study (40.06%). Following the *stating the finding* topic, the next most prevalent topic was the *nicotine patch follow-up study* (30.97%).

**Table 2:** Topic categories for tweets related to the Miyara et al study.

Group and topics		Tweets (n=1605), n (%)	Example tweets^a^
**Stance on smoking**			
	Encouraging tobacco use	51 (3.18)	“that's hilarious so nicotine is actually good for something lol smoke em if you got em lol study finds smoking reduces chance of getting coronavirus symptoms” “you may have quit smoking too soon a study shows that nicotine addiction may play a protective role against contracting covid 19”
	Stating the finding	643 (40.06)	“a cross sectional study strongly suggests that those who smoke every day are much less likely to develop a symptomatic or severe infection with covid 19 compared with the general population” “french researchers reveal that smokers are less likely to get covid 19”
**Other sentiments**			
	Shocked or surprised	28 (1.74)	“shocking study supports smoking as preventive measure against covid19” “in surprising results and a warning from smoking a Miyara et al. study finds that nicotine may help to fight corona”
	Tobacco consequences highlighted	24 (1.50)	“french research suggests nicotine could protect against covid 19 but smoking remains biggest killer in france 75 000 people die every year from smoking related complications” “france finds smoking may help you resist covid 19 if you don t mind dying of something else in a reversal of prevailing covid 19 wisdom a Miyara et al. study appears to show smokers are less at risk from virus affirming results of an earlier chinese study”
	Tobacco industry funded	34 (2.12)	“this is insane to even suggest using substance such nicotine less likely to catch coronavirus is it tobacco companies is paying for this study” “this is based on data analysis not a controlled study and i m deeply suspicious of possible big tobacco influence but as a 63 yr old still hooked on nicotine mints i m hoping hard”
Research focus in the future: nicotine patch study		497 (30.97)	“french researchers to test nicotine patches on coronavirus patients” “the study at a major paris hospital suggests a substance in tobacco possibly nicotine may be stopping patients who smoke from catching covid 19 clinical trials of nicotine patches are awaiting the approval of the country's health authorities”
Unsorted: miscellaneous		328 (20.44)	“treat this research with caution it was my solid understanding that this virus affects smokers far worse than a non smoker as the lungs are already compromised from damage by smoking” “not gonna read this as i m not a smoker but all i can say is of course it s a Miyara et al. study”

^a^During preprocessing and before hand-coding, punctuation and capitalization were removed from the tweets to facilitate content analysis.

#### Tweet Topics Related to Versus Not Related to the Miyara et al Study

By topic comparison, both similarities and differences were seen. Regarding consistency, topics found within tweets related to the Miyara et al study and those found in tweets not related to the study showed that the majority simply stated the finding of the study; 643 out of 1605 (40.06%) tweets related to the Miyara et al study belonged to this category, compared to 155 out of 324 (47.8%) tweets not related to the study. However, while both groups contained tweets that held negative attitudes toward smoking, tweets related to the Miyara et al study discouraged tobacco use in a more comprehensive way. For example, within the 1605 tweets related to the Miyara et al study, 24 (1.50%) highlighted the risks of tobacco use and 34 (2.12%) speculated whether the study was funded by the tobacco industry. Within the 198 tweets that were not related to the study, 67 (33.8%) discouraged tobacco use, while 23 (11.6%) encouraged tobacco use. Furthermore, there were tweets that demonstrated doubts regarding the potential benefits of smoking, but many did not absolutely reject such possibilities. This suggests that users may have been open to exploring whether there was a positive effect of smoking on COVID-19 but that they proceeded with caution. Before the publication of the Miyara et al study, such rejections were frequently observed, which indicates that most users shared a neutral or more negative opinion of smoking’s influence on COVID-19 and suggests that users may have begun to think more critically about smoking’s impact on the novel coronavirus after the Miyara et al study was published. The distributions of the number of followers of Twitter users who posted either tweets related to the Miyara et al study or tweets not related to the study were both highly skewed to the left. The median number of followers of Twitter users who posted tweets related to the Miyara et al study was 585 (IQR 3407). The median number of followers of Twitter users who posted tweets that were not related to the study was 630 (IQR 2681). The number of followers of Twitter users indicated the bandwidth of outreach of those posted tweets.

## Discussion

### Principal Findings

This report presents novel findings observing a shift in attitudes toward smoking among Twitter users after publication of a Miyara et al study that reported lower rates of daily smoking among COVID-19 cases. The relatively large number of median followers of Twitter users who posted tweets related to the Miyara et al study indicated that the results of the Miyara et al study were widely disseminated. Overall, the findings suggest that this study was successfully disseminated and appears to have led to more positive attitudes toward smoking among our population. Every post that stated the main finding of the Miyara et al study, which was considered a neutral sentiment, can be looked at as an instance of spread of information from one user to other users, leading to even greater spread of the study. When comparing tweet sentiments before and after April 21, 2020, there was a significantly more positive attitude toward smoking among all tweets.

The largest percentage of tweets, from before and after April 21, 2020, and that were about the Miyara et al study, had a neutral sentiment toward smoking, including mentions of wanting more information and a sense of uncertainty regarding the study’s findings. The most prevalent tweets stated the main finding of the study and/or mentioned the nicotine patch follow-up study. The main findings of the articles tweeted about in this sample—those that were not about the Miyara et al study—reflected both the benefits and risks of smoking on COVID-19 [13,14]. People’s opinions of smoking varied a lot throughout the periods, encouraging or discouraging nicotine use. Among different studies, the up- or down-regulation effects of ACE2 receptor proteins, to which viruses bind, were frequently discussed to explain smoking’s impact on COVID-19 [14,15]. The similar distributions of the number of followers of Twitter users who posted tweets that were either related to or not related to the Miyara et al study indicated similar bandwidths of outreach of those posted tweets.

Nevertheless, differences in people’s opinions and study focuses were observed in some ways. Even among posts not directly discussing the Miyara et al study, there was still a shift to a more positive attitude toward smoking after its publication. Not necessarily shifting all others’ attitudes toward smoking from negative to positive, the release of Miyara et al study did at least waver the stances of those who opposed smoking, confirming its positive impact. This speculation was supported by a 6.82% weight decrease of tweets that were not related to Miyara et al study that advocated quitting smoking. To conclude, a different reaction pattern was seen between tweets within French and non-French groups. Beyond the study’s influence of changing people’s attitudes toward smoking, it also informs a critical thinking mindset behind how people observe the effects of smoking. For example, while people’s stances varied regarding the effect of smoking on COVID-19, more tweets (10.3%) were observed that called on people to quit smoking before April 21, 2020, compared to after (3.5%). This decrease was perhaps due to the impact of the Miyara et al study, which highlighted a potential benefit of smoking.

Significant changes were also seen regarding how people analyzed the impacts of smoking on COVID-19. Before April 21, 2020, many tweets (14.3%) discussed the increased risks of infection if smokers had existing health conditions, such as diabetes and asthma [16]. Some tweets cited articles that stated former smokers would be at higher risks of infection compared to current smokers [17]. These mixed perspectives did not analyze the direct impact of smoking and reiterated the combined effects of existing health issues and smoking on COVID-19 infection. The findings of these articles reflect the uncertainty of research directions about smoking itself, which was expressed in the tweets that were not related to the Miyara et al study. However, the publication of the Miyara et al study could have led to less uncertainty about the impact of smoking on COVID-19 after April 21, 2020, with only 1.5% of tweets discussing the influence of smokers’ health conditions on infection, compared to 14.3% before.

### Strengths

Twitter is a valuable tool used in health research and can be used to analyze up-to-date data about a specific topic while it is at its peak discussion point [18]. Another study examining Twitter sentiments on smoking and COVID-19 found that the sentiment of tweets was generally negative but became less negative in April 2020, which is when the Miyara et al study was released [11]. We observed a shift toward positive sentiments revolving around smoking among posts discussing the Miyara et al study after April 21, 2020, compared to posts before the publication. Overall, this study presents a useful example of the impact of the dissemination of a particular contrarian study and how it can shift the field of discussion on a topic. That is, one particular finding can color a conversation.

### Limitations

Although the results met our original expectations of the impact of the Miyara et al study on people’s opinion changes, several limitations of our analysis can be found. Firstly, some posts might be missing, since keywords were used to filter out tweets before hand-coding. The potential problems of this are whether or not all posts about our topic contained the chosen keywords. For example, the keywords “study” and “research” were used to filter out research-related posts; however, users might use words like “result” and “report,” among other words, to reflect a research finding. In our case, “result” and “report” would bring in a lot of off-topic tweets; thus, those were not included in the keyword list. Nevertheless, an alternative analysis could consider adding those tweets and starting the filtering process afterward. Similar problems might be relevant if some users comment on a research result without referring to the subject, in which case users’ sentiments would still be related to our analysis but would be ignored. Secondly, we analyzed tweets written only in English and missed tweets in other languages, which might bias the study results. However, analyzing tweets written only in English could avoid misinterpreting translations. Thirdly, we did not collect and analyze the numbers of likes and retweets of the original posts, which could also help to analyze the impact of those tweets. Furthermore, although intercoder variances were small enough to make the results valid, hand-coding is a subjective method, implying potential cognitive differences in coding. Tweets were posted globally, which suggests that tones from distinct regions might imply different meanings (eg, sarcasm). Though hard to eliminate, such limitations could be alleviated by incorporating more coders with diverse backgrounds. Lastly, it is possible that smokers tended to tweet more about smoking-related studies. They may even have had positive attitudes toward smoking before seeing any studies demonstrating smoking’s beneficial effects. To address that, studies quantifying such likelihood may be needed and should be taken into consideration during analysis to more precisely observe the impact of the Miyara et al study.

### Implications and Future Directions

Note that this paper is not a criticism of the Miyara et al study’s authors or their research—our focus here is on using that paper and its findings as a jumping-off point for exploring how a particular study is disseminated on Twitter, and how that information may influence the sentiment of tweets moving forward. Information-based communication strategies can be used to modify people’s attitudes by providing evidence for or against an idea. Previous literature has described the impact of research and research dissemination as affecting knowledge, attitudes, and behavior with respect to health risks [19]. Our findings suggest that among Twitter users discussing tobacco research, a substantial number of posts were related to the Miyara et al study on COVID-19 and smoking for several days after its publication on April 21, 2020. There was a significant increase in the number of tweets with a positive sentiment toward smoking, both when comparing tweets posted before and after the Miyara et al study publication date and when comparing tweets related to and not related to the Miyara et al study, after April 21, 2020. Therefore, the results of the Miyara et al study could have contributed to a positive shift in attitude toward smoking among some Twitter users. We understand the number of tweets used in the analysis might be only 1% of the total number of tweets related to the Miyara et al study on COVID-19. However, given the assumption that the tweets obtained from free Twitter streaming could be treated as a random sample from all tweets related to the Miyara et al study on COVID-19, the statistical significance of a positive shift in attitude toward smoking is valid. Given the negative consequences of tobacco use, it is imperative to disseminate accurate messaging and concise evidence and recommendations regarding the relationship between COVID-19 and nicotine to prevent initiation of tobacco product use and encourage cessation. Given the dissemination of the Miyara et al study’s results and the confusion expressed by users, there is a need for further research on the true effects of nicotine and the novel coronavirus. The World Health Organization has since published a scientific brief stating that “smoking is associated with increased severity of disease and death in hospitalized COVID-19 patients” and that “there is no evidence to quantify the risk to smokers of hospitalization with COVID-19 or of infection by SARS-CoV-2” [20]. Twitter can serve as a useful resource to monitor the spread of, and reactions to, tobacco research to identify potentially problematic public interpretations or misrepresentations of findings.

## Abbreviations

ACE2: angiotensin-converting enzyme 2
FDA: US Food and Drug Administration
NIH: National Institutes of Health
